# Cutaneous metastases from rectal adenocarcinoma: A case report

**DOI:** 10.1177/2050313X241309093

**Published:** 2024-12-20

**Authors:** Samia Rahman, Parbeer Grewal

**Affiliations:** Department of Medicine, University of Alberta, Edmonton, AB, Canada

**Keywords:** Cutaneous metastases, rectal adenocarcinoma

## Abstract

Cutaneous metastases from colorectal cancer are an uncommon but critical finding, typically signaling advanced disease with poor prognosis. This case report describes a 64-year-old woman with a limited past medical history who presented to our outpatient dermatology practice with rapidly spreading erythematous, indurated, and nearly verruciform plaques in the groin, vaginal, and perineal region. Biopsy confirmed metastatic adenocarcinoma of colonic origin, and diagnostic imaging, and colonoscopy revealed stage IV colorectal cancer involving extensive cutaneous, lymphatic, and visceral metastases. Unfortunately, the patient had not received routine colorectal cancer screening, despite a positive family history. Due to extensive disease, palliative radiation was not an option, and systemic chemotherapy was initiated. This case emphasizes the need for awareness of cutaneous metastases as a potential initial presentation of undiagnosed malignancies, the importance of routine colorectal cancer screening, and timely biopsy of suspicious skin lesions for early diagnosis and management.

## Introduction

Cutaneous metastases from colorectal cancer (CRC) are an uncommon but significant clinical finding, typically indicating advanced, disseminated disease with poor prognosis.^1–3^ These metastases can present in a variety of forms, often mimicking benign skin conditions, which can delay diagnosis and treatment.^4,5^ Understanding the diverse presentations of cutaneous metastases in CRC is crucial for timely identification and appropriate management, particularly given the high mortality rate associated with metastatic CRC.^3–5^

## Case report

A 64-year-old Caucasian female patient presented to our community dermatology practice with a 1-month history of a “rash” in her genital area and buttocks that was rapidly worsening and spreading. She also endorsed a 6-month history of increasing erythema, irritation, and pruritus in the genital and perianal region causing discomfort when ambulating and sitting. She initially assumed it was an allergic reaction and tried over-the-counter oral and topical anti-allergens. Upon further discussion, the patient endorsed changes in bowel habits with alternating constipation and diarrhea and increasingly string-like stools, as well as weight loss and dysuria. The patient had a limited notable past medical history, with a remote history of lichen sclerosus and no regular medications. She had a family history of CRC with her father being diagnosed with colon cancer at 86 years old. Based on local cancer screening guidelines, the patient was in the moderate risk category for CRC and should have received fecal immunochemical testing (FIT) every 1–2 years starting at age 40. Unfortunately, the patient only received fecal occult blood testing at age 50 and FIT testing at 55—both of which were negative.

On physical examination, the patient presented with irregular, erythematous plaques in the vaginal, inguinal, perineal, and perianal regions, extending into the vaginal canal. These plaques were lichenified and indurated with accentuated skin markings and occasional areas of excoriation. Many of these plaques showed an almost verruciform, hyperkeratotic, and thickening of the skin.

A shave biopsy of the right inguinal fold was consistent with metastatic adenocarcinoma. There was prominent necrosis with heavy neutrophil infiltration and prominent positive staining to EMA, CEA, CK20, and CX2, thus consistent with colonic origin.

Positron emission tomography, computed tomography, magnetic resonance imaging scans, and a colonoscopy confirmed stage IV CRC. The investigations showed a 12.3 cm invasive rectal adenocarcinoma (low grade with normal MMR, p53 wild-type, and p16 negative) infiltrating through the perianal skin on the right side and extending anteriorly into the labia majora, with metastasis of extensive contiguous superficial perianal, perineal, and bilateral inguinal dermal involvement. There was also inguinal, pelvic, mediastinal, cervical, and hilar lymphadenopathy along with hepatic, adrenal, thyroid, and pleural metastasis. With these investigations, she was also incidentally found to have a second primary tumor of biopsy-proven endometrial adenocarcinoma.

**Figure 1.**
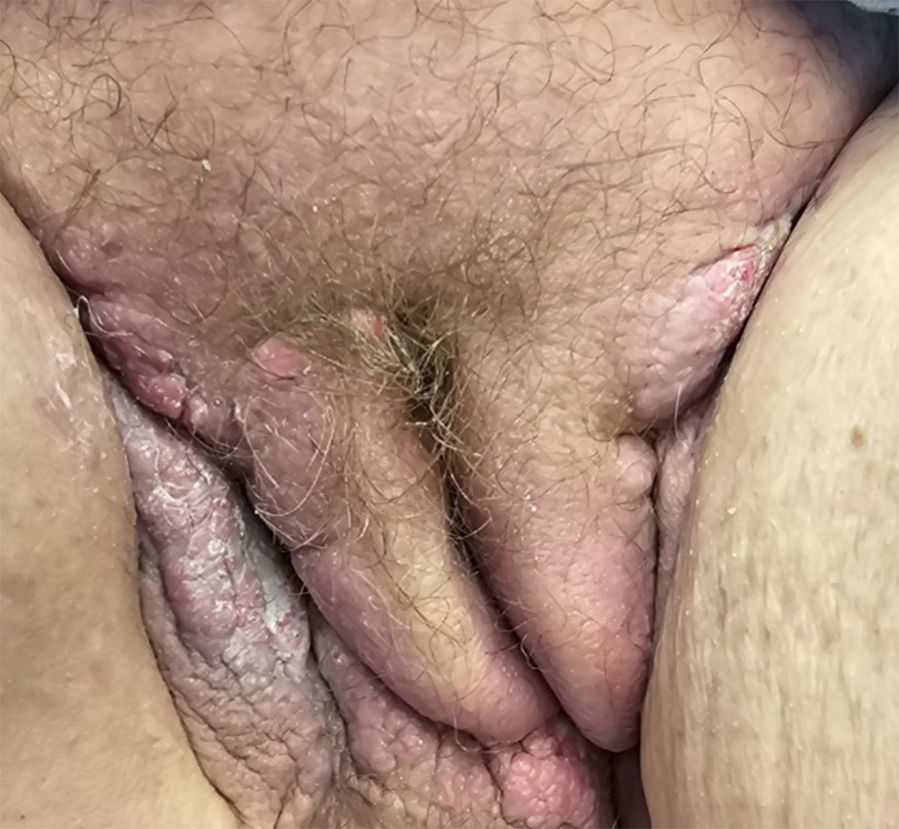
Perineal, bilateral inguinal, and genital cutaneous metastases.

Given the extensive spread of her cancer, it was not feasible to include all affected areas within the radiation field, rendering her ineligible for palliative radiation or surgery. Consequently, systemic chemotherapy with FOLFIRI was determined to be the most appropriate course of treatment (Figure 1).

## Discussion

Although cutaneous metastasis from CRC is rare, occurring in less than 4% of cases, it is a critical indicator of disseminated disease and is associated with a very poor prognosis.^6^ Over two-thirds of patients with cutaneous metastases succumb to the disease within 6 months of diagnosis.^7^ In a study by Schoenlaub et al., the median survival period for patients with cutaneous metastases originating from colorectal tumors was reported to be only 4.4 months.^8^

Cutaneous metastases from internal malignancies can manifest in a variety of clinical presentations, most commonly as firm, non-painful nodules that are rubbery or hard in texture and vary in size and color.^9,10^ These nodules often appear suddenly, grow rapidly, and may be mobile or fixed, discrete or multiple, and range in color from flesh-toned to violaceous or blue-black.^11^ In addition to nodules, cutaneous metastases can present as papules, plaques, ulcers, bullae, or cellulitis-like lesions, with the specific pattern of skin involvement often depending on the primary cancer.^12^ For example, rectal carcinoma cutaneous metastases have no distinctive features, but most often present as small, coalescing subcutaneous or intradermal nodules, typically measuring less than 5 cm in diameter.^13^ These lesions are frequently located near the primary tumor site and may be asymptomatic, though they can also be associated with pain, tenderness, or pruritus. Due to the wide variety of presentations and the potential for these lesions to mimic benign dermatological conditions such as cysts, lipomas, granulomas, and neurofibromas, a high index of clinical suspicion and biopsy of suspicious lesions is essential.^9^ This is particularly important as cutaneous metastases can sometimes be the first indication of an undiagnosed internal malignancy, making timely biopsy and diagnosis crucial. The majority of cases (>70%) of cutaneous metastases involve a prior history of malignancy, with the average interval between diagnosis of primary tumor and development of metastasis being approximately 2 years.^14^ However, as illustrated in our case, some patients may develop these skin metastases with minimal/non-specific visceral symptoms and limited comorbidities.

This case highlights the significance of cutaneous metastases as a marker of advanced CRC and underscores the need for routine CRC screening and prompt recognition of cutaneous metastases due to their poor prognostic implications. Given the diverse presentations of these metastases, clinicians should maintain a high index of suspicion and biopsy of suspicious skin lesions, particularly in patients with a history or risk of CRC.
